# Prospective Evaluation of CEUS-URM in Axillary Lymph Nodes with Diffuse Cortical Thickening in Breast Cancer Patients

**DOI:** 10.3390/medsci14030392

**Published:** 2026-07-14

**Authors:** Roxana Pintican, Calin Schiau, Nicoleta Antone, Filip Madalina, Andrei Roman, Carmen Lisencu, Ovidiu Balacescu, Vlad-Alexandru Gata, Maximilian Vlad Muntean, Patriciu Achimas-Cadariu

**Affiliations:** 1 Department of Radiology, Prof. Dr Ion Chiricuta Oncology Institute, 400015 Cluj-Napoca, Romania; roxana.pintican@gmail.com (R.P.); andrei.roman678@gmail.com (A.R.); carmen_lisencu@yahoo.com (C.L.); 2 Department of Radiology, “Iuliu Hatieganu” University of Medicine and Pharmacy, 400012 Cluj-Napoca, Romania; madalina.salajan14@gmail.com; 3 Department of Radiology, Emergency County Hospital, 400006 Cluj-Napoca, Romania; 4 Department of Oncological Surgery and Oncological Gynecology, “Iuliu Hatieganu” University of Medicine and Pharmacy, 400012 Cluj-Napoca, Romania; 5 Department of Genetics, Genomics and Experimental Pathology, Prof. Dr Ion Chiricuta Oncology Institute, 400015 Cluj-Napoca, Romania; obalacescu@yahoo.com; 6 Department of Surgery, Prof. Dr Ion Chiricuta Oncology Institute, 400015 Cluj-Napoca, Romania; gatavlad@yahoo.com (V.-A.G.); maximilian.muntean@gmail.com (M.V.M.); 7 Department of Surgical Oncology, “Iuliu Hatieganu” University of Medicine and Pharmacy, 400012 Cluj-Napoca, Romania

**Keywords:** CEUS, microvascular imaging, metastatic nodes, breast cancer, axilla

## Abstract

**Background**: Conventional axillary ultrasound (US) is least reliable in lymph nodes with diffusely thickened cortex. We evaluated whether contrast-enhanced ultrasound with ultra-resolution microvascular imaging (CEUS-URM) improves discrimination of metastatic nodes in this subgroup. **Materials and methods**: This was a prospective single-center study of patients with histologically confirmed breast cancer; one index (most suspicious) node per patient (unit of analysis = index node). Two separately recruited consecutive cohorts were analyzed: a US-only cohort (*n* = 181; diffuse-thickening subgroup with histology, *n* = 52) and a subsequent CEUS-URM cohort (*n* = 42; 15 metastatic). Surgical histopathology (SLNB/ALND) was the reference standard. A CEUS-URM score was built from data-driven, Youden-optimized cut-offs (URM vessel count ≥8; DV mean density ≥ 13.05) and internally validated by bootstrap with optimism correction. As the cohorts were separate, the US-versus-CEUS comparison is cross-cohort and exploratory. **Results**: Within the diagnostically challenging subgroup of lymph nodes with diffusely thickened cortex, conventional axillary US alone demonstrated limited discriminatory performance for metastatic involvement (AUC 0.43). CEUS-derived quantitative parameters significantly improved diagnostic accuracy, with the best individual parameter achieving an AUC of 0.68. A simple CEUS score combining hypervascular vessel count and vascular density provided the highest diagnostic performance (AUC 0.81, 95% CI 0.68–0.92). At a low threshold, the CEUS score showed high sensitivity (93%), suitable for screening and exclusion of nodal metastases, while at a higher threshold it achieved high specificity (96%), allowing reliable confirmation of metastatic disease. **Conclusions**: In this exploratory study, a simple CEUS-URM score improved discrimination of diffusely thickened axillary nodes and may serve as an adjunct to conventional US. The findings are preliminary—derived and tested in the same small cohort—and require external, within-patient paired validation.

## 1. Introduction

Breast cancer remains the most frequently diagnosed malignancy among women worldwide and a major cause of cancer-related morbidity and mortality. Accurate assessment of axillary lymph node status is central to staging, prognosis, and treatment planning, influencing decisions regarding surgery, radiotherapy, and systemic therapy [1,2,3]. Over the past two decades, axillary management has shifted from routine axillary lymph node dissection toward less invasive approaches, including sentinel lymph node biopsy and selective axillary treatment, in order to reduce morbidity without compromising oncologic safety [4,5,6,7,8,9].

Conventional axillary ultrasound is widely used as a first-line imaging tool for preoperative nodal assessment because it is accessible, non-invasive, and allows image-guided biopsy of suspicious lymph nodes. However, its diagnostic performance is variable and depends heavily on morphologic criteria such as cortical thickness, cortical asymmetry, shape, and hilar preservation [10,11,12,13,14,15,16]. In particular, lymph nodes with diffusely thickened cortex represent a diagnostic challenge, as this pattern may occur in both reactive and metastatic nodes, reducing the discriminatory value of conventional B-mode ultrasound.

Contrast-enhanced ultrasound (CEUS) provides dynamic information regarding nodal perfusion and microvascular architecture and has shown promising results for sentinel and axillary lymph node evaluation in breast cancer [17,18,19,20,21,22,23,24,25,26]. More recently, super-resolution and ultra-resolution microvascular imaging techniques (URM) have enabled visualization and quantification of microvascular structures beyond the limits of conventional Doppler imaging [27,28,29,30]. Nevertheless, evidence remains limited regarding the added value of CEUS-URM specifically in axillary lymph nodes with diffuse cortical thickening, where conventional ultrasound performs least reliably.

Therefore, this study aimed to evaluate whether the addition of CEUS-URM improves the diagnostic performance for detecting metastatic lymph nodes in breast cancer patients presenting with diffusely thickened nodal cortex. We further assessed whether quantitative CEUS-URM parameters and a simple CEUS-URM score could improve discrimination between benign and metastatic lymph nodes.

## 2. Materials and Methods

### 2.1. Study Design and Population

**Figure 1:**
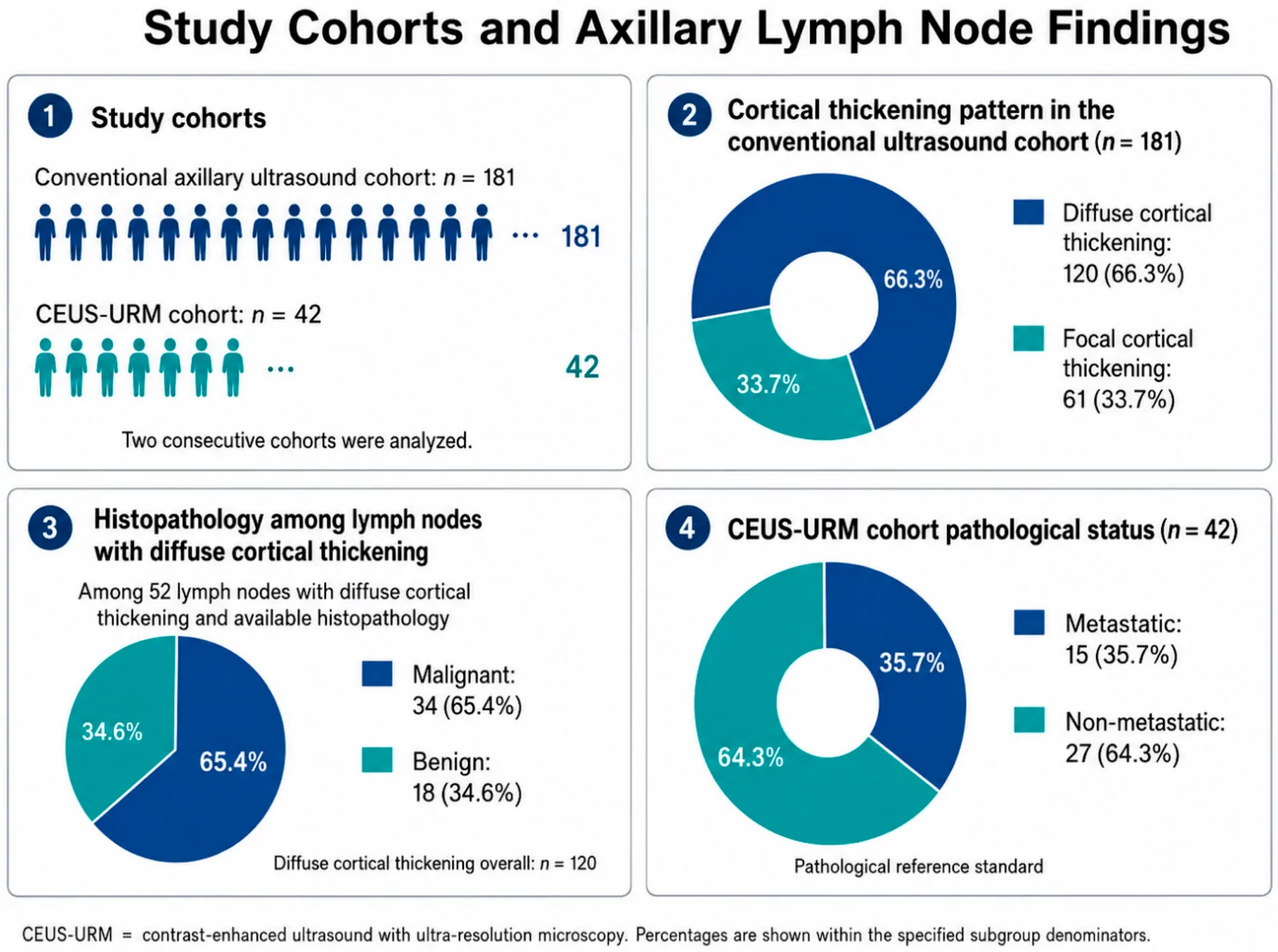
Study population.

This prospective, institutional review board-approved study included patients with breast cancer referred for preoperative axillary evaluation (approval no. 305/30.08.2025). Inclusion criteria consisted of histologically confirmed breast cancer patients, without neoadjuvant treatment or previous surgery on the breast/axilla, and indication for surgery. Patients were enrolled consecutively within each study period, and identical inclusion and exclusion criteria were applied throughout; the two cohorts differed only in the ultrasound platform available during their respective periods. A single index (most suspicious) lymph node was assessed per patient, so the unit of analysis was the index lymph node (one node per patient). The flow of participants, including post-enrolment exclusions, is summarized in Figure 1. Two consecutive cohorts were included. The first cohort included patients evaluated with conventional axillary ultrasound alone, whereas the second cohort included patients examined using CEUS-URM. All patients with suspicious lymph nodes (cortical thickness ≥ 3 mm, no fatty hilum, round shape and/or blurred margins) underwent a core-needle biopsy (CNB; 14 G needle). Patients with axillary lymph nodes negative on CNB subsequently underwent sentinel lymph node biopsy, with one to three sentinel lymph nodes excised. Conversely, patients with axillary lymph nodes positive on CNB underwent axillary lymph node dissection. Thus, the final nodal status was always established by surgical histopathology (sentinel-node biopsy or axillary dissection), with CNB used only as a triage test and never as the sole reference standard. Lymph nodes were classified as benign or metastatic based on the final pathological result. Exclusion criteria consisted of patients without pathological verification or incomplete imaging (ultrasound or CEUS-URM). A single index (most suspicious) lymph node was assessed per patient; the unit of analysis was therefore the index lymph node (one node per patient).

### 2.2. Conventional Axillary Ultrasound

All patients underwent standard axillary ultrasound examination using high-frequency linear-array transducer from Samsung RS85 Prestige (Samsung Medison Co., Ltd., Hongcheon-gun, Gangwon-do, Republic of Korea, 2024). Lymph nodes were assessed in grayscale mode and Color-Doppler evaluation. Morphologic criteria included cortical thickness, cortical symmetry, nodal shape, preservation or loss of the fatty hilum, and vascular pattern. Particular attention was given to lymph nodes with diffuse cortical thickening, defined as generalized, concentric cortical enlargement (cortical thickness ≥ 3 mm) involving the whole node rather than focal cortical bulging or eccentric cortical lobulation, in line with previously described morphologic criteria [12,13].

### 2.3. CEUS-URM Technique

In the CEUS-URM cohort, conventional ultrasound was performed before contrast injection and served as the baseline examination for subsequent CEUS-URM analysis. The most suspicious lymph node was evaluated after contrast. CEUS-URM examinations were performed using a high-end VINNO ultrasound system equipped with Ultra-Resolution Microvasculature imaging (URM) technology (VINNO Technology [Suzhou] Co., Ltd., Suzhou, China). URM is a contrast-enhanced ultrasound-based technique designed to visualize and quantify microvascular architecture beyond the spatial resolution limits of conventional Doppler imaging. Following intravenous administration of an ultrasound contrast agent, CEUS-URM applies advanced signal processing and microbubble tracking algorithms to suppress tissue background signals and enhance microvascular flow signals. This allows reconstruction of microvascular networks within a predefined region of interest. This technology is relevant because it provides information not only on the presence of vascularity, but also on the spatial organization and density of microvascular structures.

For CEUS-URM examination, 1.5 mL of SonoVue was administered intravenously, followed by a saline flush. Images were acquired immediately after contrast injection and continuously recorded for 120 s. The lymph node cortex was evaluated dynamically, and regions of interest were placed within visually identified hypervascular and hypovascular cortical areas. Quantitative parameters were extracted from the hypervascular region of interest and included URM vessel count, URM mean distance, URM maximum diameter, DV mean density, PI percentage, PI mean, and PI sum. In metastatic lymph nodes, paired comparisons between hypo- and hypervascular regions were also performed to assess intranodal vascular heterogeneity.

### 2.4. CEUS-URM Score

In addition to individual quantitative parameters, a simple CEUS-URM score was generated using hypervascular features, particularly vessel count and vascular density. This score was designed as a pragmatic tool to integrate microvascular burden into a single diagnostic measure. The diagnostic performance of the CEUS-URM score was compared with conventional ultrasound alone and with individual CEUS-URM parameters.

### 2.5. Statistical Analysis

Continuous variables were summarized as median and interquartile range. Between-group comparisons of CEUS-URM parameters in benign versus metastatic lymph nodes were performed using the two-sided Mann–Whitney U test. Effect sizes were calculated using the rank-biserial correlation coefficient and interpreted as small, moderate, or large. To account for multiple testing, *p*-values were adjusted using the Holm method. A secondary Holm correction was also applied to a predefined subset of clinically prioritized parameters, including URM vessel count, DV mean density, PI percentage, and PI mean.

In the US-only cohort, conventional diagnostic performance was assessed by ROC analysis of cortical thickness against the surgical reference standard, restricted to the diffuse-cortical-thickening subgroup (52 nodes with histology; 34 malignant, 18 benign). Within this deliberately selected subgroup—where every node already shows a thickened cortex—the morphologic criterion was non-discriminatory (AUC 0.43), in contrast to literature values obtained in unselected axillae comprising nodes of all morphologies.

For paired comparisons between hypovascular and hypervascular regions within metastatic lymph nodes, the Wilcoxon signed-rank test was used. Diagnostic performance was assessed by calculating sensitivity, specificity, positive predictive value, negative predictive value, and ROC-derived area under the curve. Exact 95% confidence intervals were calculated for diagnostic performance metrics. Internal validation was performed using bootstrap resampling with 10,000 iterations.

A CEUS-URM score was constructed using two data-driven hypervascular features: URM vessel count and DV mean density. One point was assigned for URM vessel count ≥8 and one point for DV mean density ≥ 13.05, yielding a score ranging from 0 to 2. These cut-offs were identified in the present cohort using the Youden index and are therefore optimized rather than externally pre-specified; because they were derived and tested on the same data, internal validation was performed by bootstrap with optimism correction, and external validation is required before clinical use. Diagnostic performance was assessed using receiver operating characteristic analysis, with AUCs reported together with bootstrap 95% confidence intervals. Paired comparisons between the CEUS-URM score and individual CEUS-URM parameters were performed using the DeLong test and confirmed by paired bootstrap resampling.

Threshold-specific sensitivity, specificity, positive predictive value, negative predictive value, accuracy, and likelihood ratios were calculated with exact binomial 95% confidence intervals. To account for the small sample size and sparse diagnostic cells, Firth penalized logistic regression was used to estimate the association between the CEUS-URM score and metastatic lymph node status. Internal validation was performed using bootstrap resampling, including optimism correction for the score AUC. Decision curve analysis was performed to estimate the clinical net benefit of the CEUS-URM score across a range of threshold probabilities.

The pre-specified primary endpoint was the AUC of the CEUS-URM score for metastatic involvement; the seven individual quantitative parameters were analyzed as exploratory endpoints with Holm correction for multiplicity. In the US-only cohort, conventional diagnostic performance was assessed by ROC analysis of cortical thickness against the surgical reference standard, restricted to the diffuse-cortical-thickening subgroup (52 nodes with histology; 34 malignant, 18 benign); this subgroup-specific value is not directly comparable to literature figures obtained in unselected axillae comprising nodes of all morphologies.

## 3. Results

### 3.1. Study Population

Two consecutive cohorts were analyzed. The initial cohort included 181 patients evaluated with conventional axillary ultrasound alone, whereas the subsequent cohort included 42 patients assessed using CEUS-URM. Diffuse cortical thickening was observed in 120 lymph nodes (66.3%), whereas focal cortical thickening was observed in 61 lymph nodes (33.7%), out of a total of 181 lymph nodes. Among 52 lymph nodes with diffuse cortical thickening and available histopathology, 34 were malignant (65.4%) and 18 were benign (34.6%). In the CEUS-URM cohort, 15 of 42 lymph nodes were metastatic according to the pathological reference standard, corresponding to a positivity rate of 35.7%. Of these 42 index nodes (15 metastatic, 27 benign), complete quantitative CEUS-URM parameters were available in 41 (one benign node had incomplete quantification); accordingly, the qualitative combined-read 2 × 2 analysis included all 42 nodes, whereas the quantitative-parameter and score analyses were based on the 41 complete cases (15 metastatic, 26 benign) (Figure 1).

The mean age of the patients was 46 years (range: 28–79 years). The distribution of molecular subtypes was comparable between the CEUS and US groups. HR+/HER2− tumors were the most frequent subtype in both groups, accounting for 82.9% of cases in the CEUS group and 74.7% in the US group. Baseline clinicopathological characteristics were comparable between the US-only and CEUS cohorts. No statistically significant differences were observed regarding age, tumor size category, cortical thickness of the evaluated lymph node, histological Nottingham grade, or molecular subtype distribution, supporting the comparability of the two cohorts (all *p* > 0.05) (Table 1).

### 3.2. Quantitative CEUS-URM Parameters

Quantitative CEUS-URM analysis was performed within the hypervascular region of interest of the diffusely thickened nodal cortex. Malignant lymph nodes showed a higher URM vessel count than benign nodes, with median values of 11.00 and 7.00, respectively. This difference showed a moderate effect size but did not remain statistically significant after Holm correction. DV mean density and perfusion-intensity-derived parameters also tended to be higher in metastatic lymph nodes, suggesting increased microvascular density and perfusion; however, these differences were associated with small effect sizes and did not reach statistical significance after correction for multiple comparisons. Overall, individual CEUS-URM parameters showed modest discriminative performance, with AUC values ranging from 0.41 to 0.68; URM vessel count showed the highest individual AUC (Table 2).

**Figure 2:**
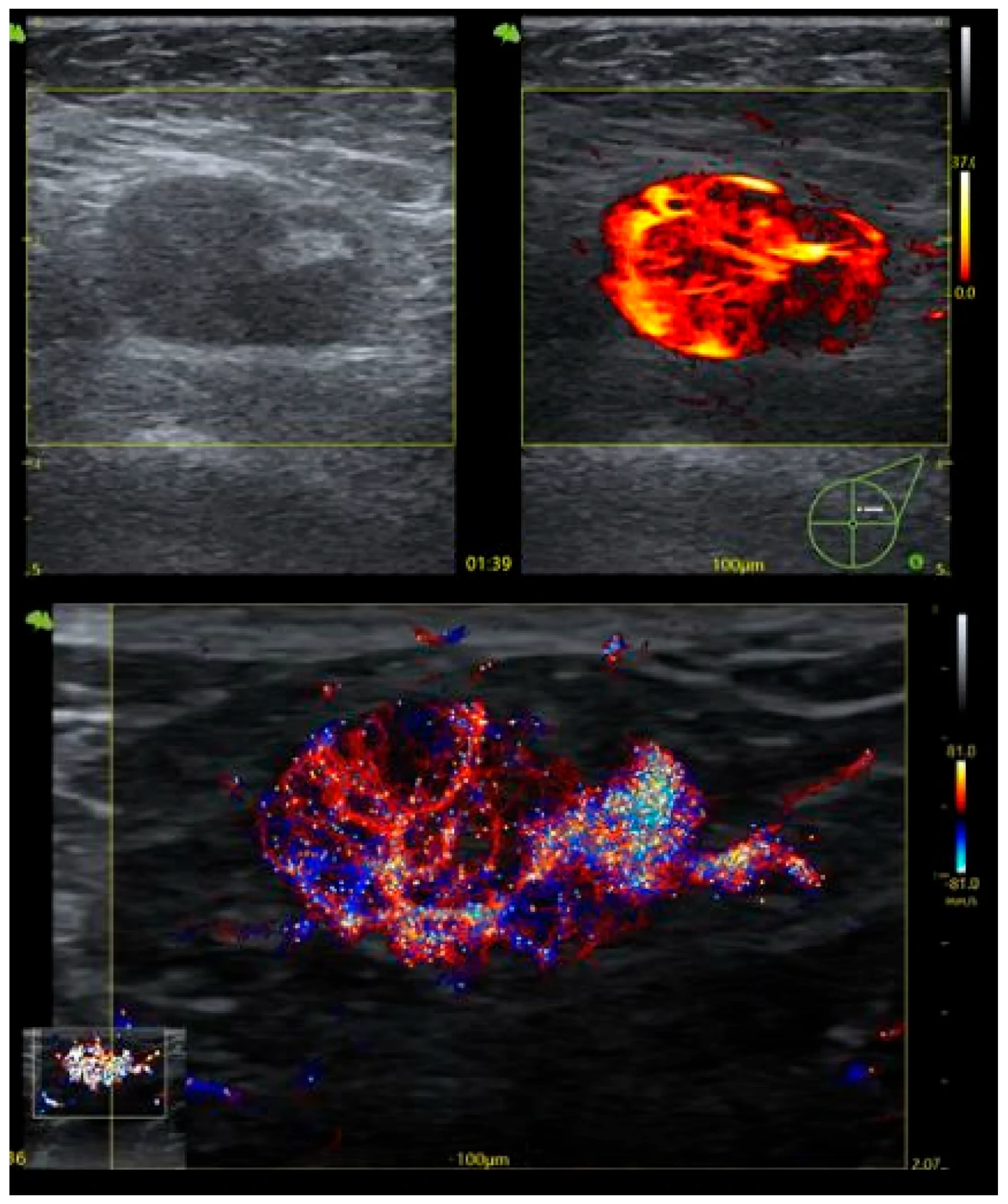
Metastatic axillary lymph node showing grayscale ultrasound and CEUS-URM assessment of hypo- and hypervascular cortical areas.

A representative metastatic lymph node is shown in Figure 2, illustrating grayscale ultrasound and CEUS-URM assessment of hypo- and hypervascular areas within the diffusely thickened cortex.

### 3.3. ROC Analysis of Individual CEUS-URM Parameters

**Figure 3:**
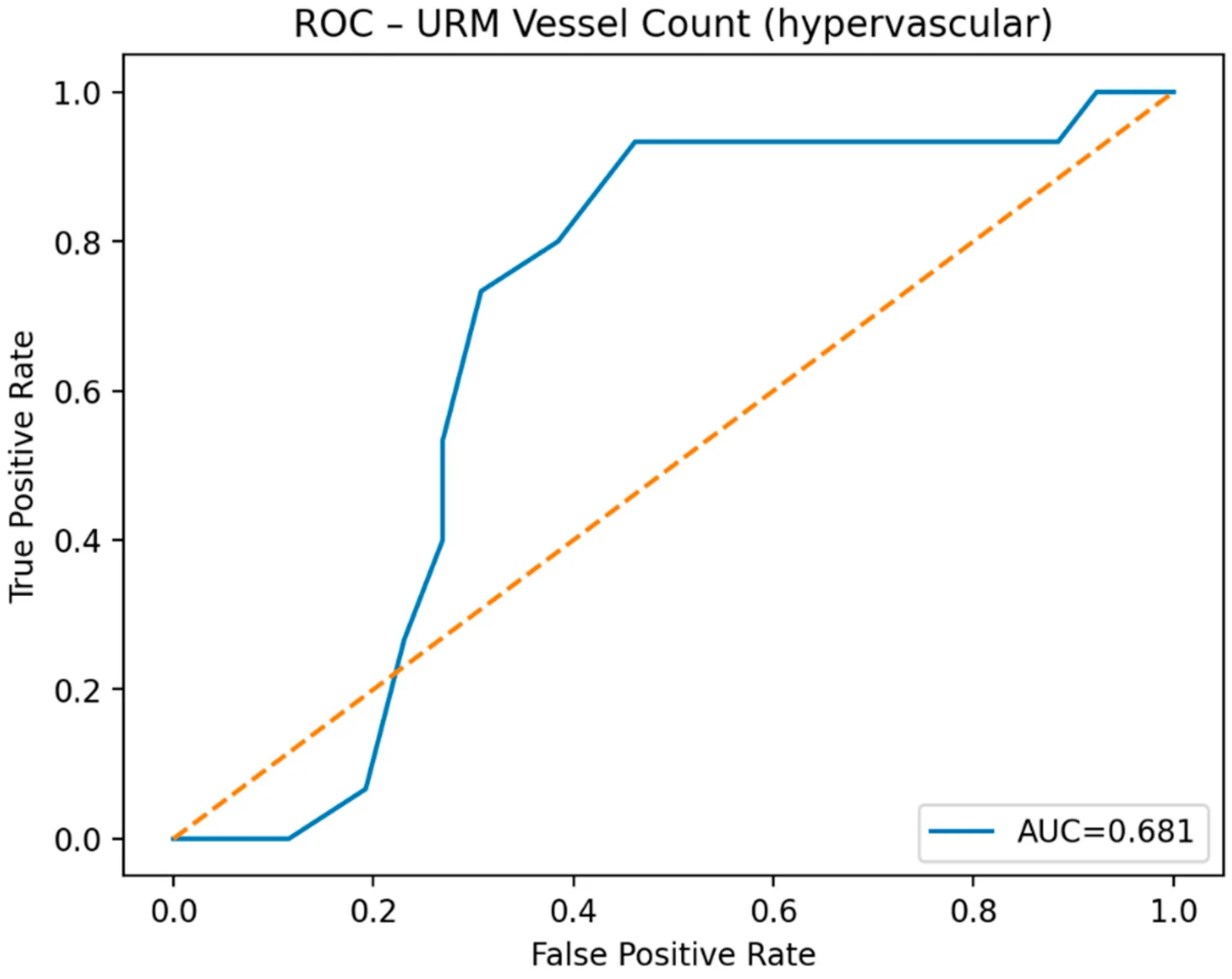
ROC curve of URM vessel count for predicting axillary lymph node metastasis.

**Figure 4:**
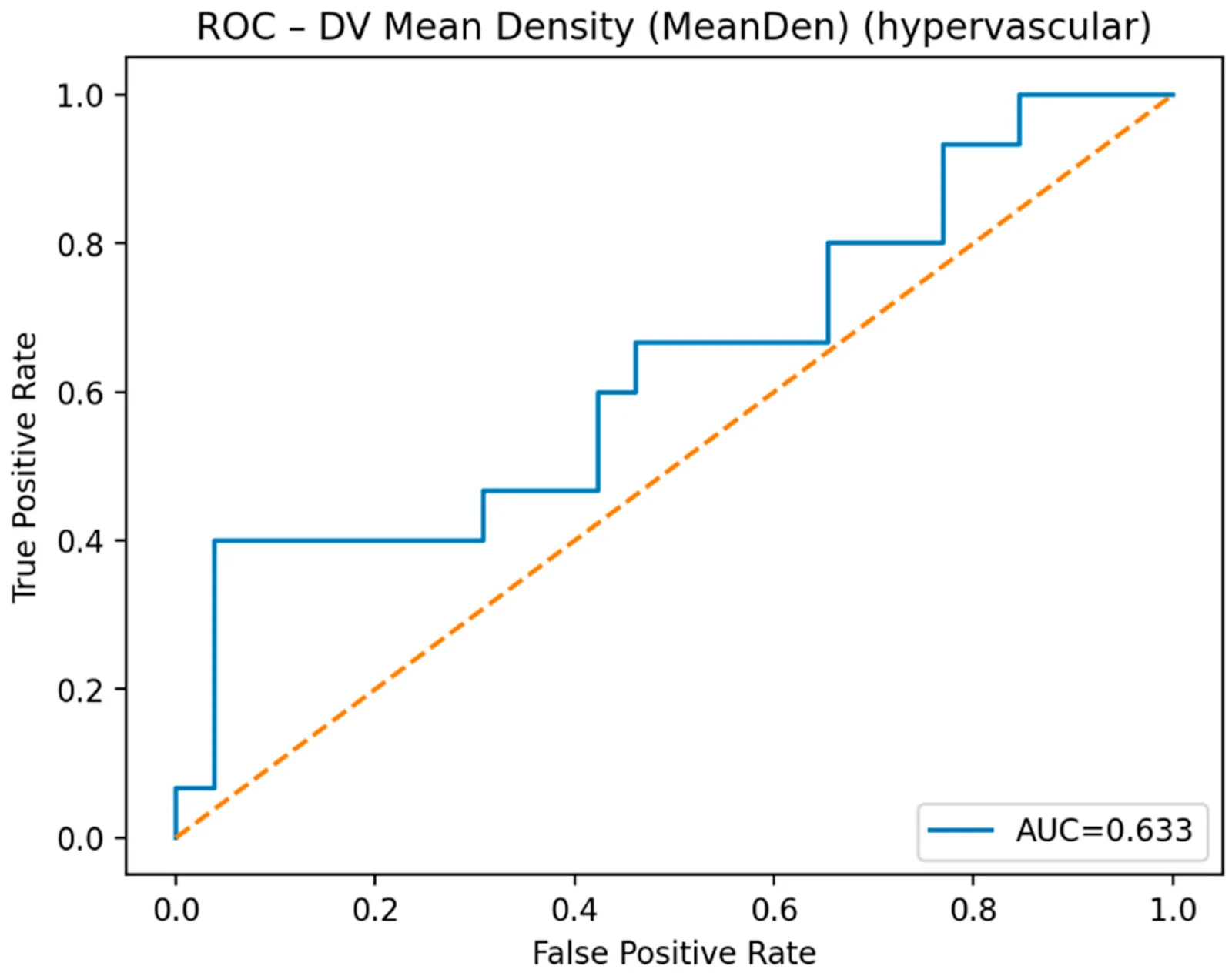
ROC curve of DV mean density for predicting axillary lymph node metastasis.

ROC analysis showed modest discriminative performance for individual CEUS-URM parameters. Among the analyzed hypervascular parameters, URM vessel count provided the highest individual diagnostic performance, with an AUC of 0.681 (Figure 3). DV mean density showed lower discriminative ability, with an AUC of 0.633 (Figure 4). Other quantitative parameters, including URM mean distance, maximum diameter, and perfusion-intensity metrics, showed limited diagnostic performance, with AUC values ranging from poor to moderate.

### 3.4. Diagnostic Performance of CEUS-URM

Using the combined US + CEUS-URM approach, 15 true-positive, 18 true-negative, nine false-positive, and no false-negative results were observed. This corresponded to a sensitivity of 100% (exact 95% CI: 78.2–100%), specificity of 66.7% (exact 95% CI: 46.0–83.5%), positive predictive value of 62.5% (exact 95% CI: 40.6–81.2%), and negative predictive value of 100% (exact 95% CI: 81.5–100%). Bootstrap-derived confidence intervals were comparable to the exact intervals, supporting the internal robustness of these estimates despite the limited sample size (Table 3 and Figure 2).

### 3.5. CEUS-URM Score

The CEUS-URM score was calculated in 41 complete cases, including 15 metastatic and 26 benign lymph nodes. The score achieved an AUC of 0.810 (bootstrap 95% CI: 0.673–0.916), exceeding the diagnostic performance of URM vessel count alone (AUC 0.681, bootstrap 95% CI: 0.498–0.849) and DV mean density alone (AUC 0.633, bootstrap 95% CI: 0.444–0.810). In paired DeLong analyses performed within the complete-case CEUS-URM cohort, the score showed significantly higher AUC than URM vessel count alone (AUC difference 0.129; *p* = 0.033) and DV mean density alone (AUC difference 0.177; *p* = 0.004). Bootstrap optimism correction yielded an optimism-corrected AUC of 0.773, supporting moderate internal robustness.

**Figure 5:**
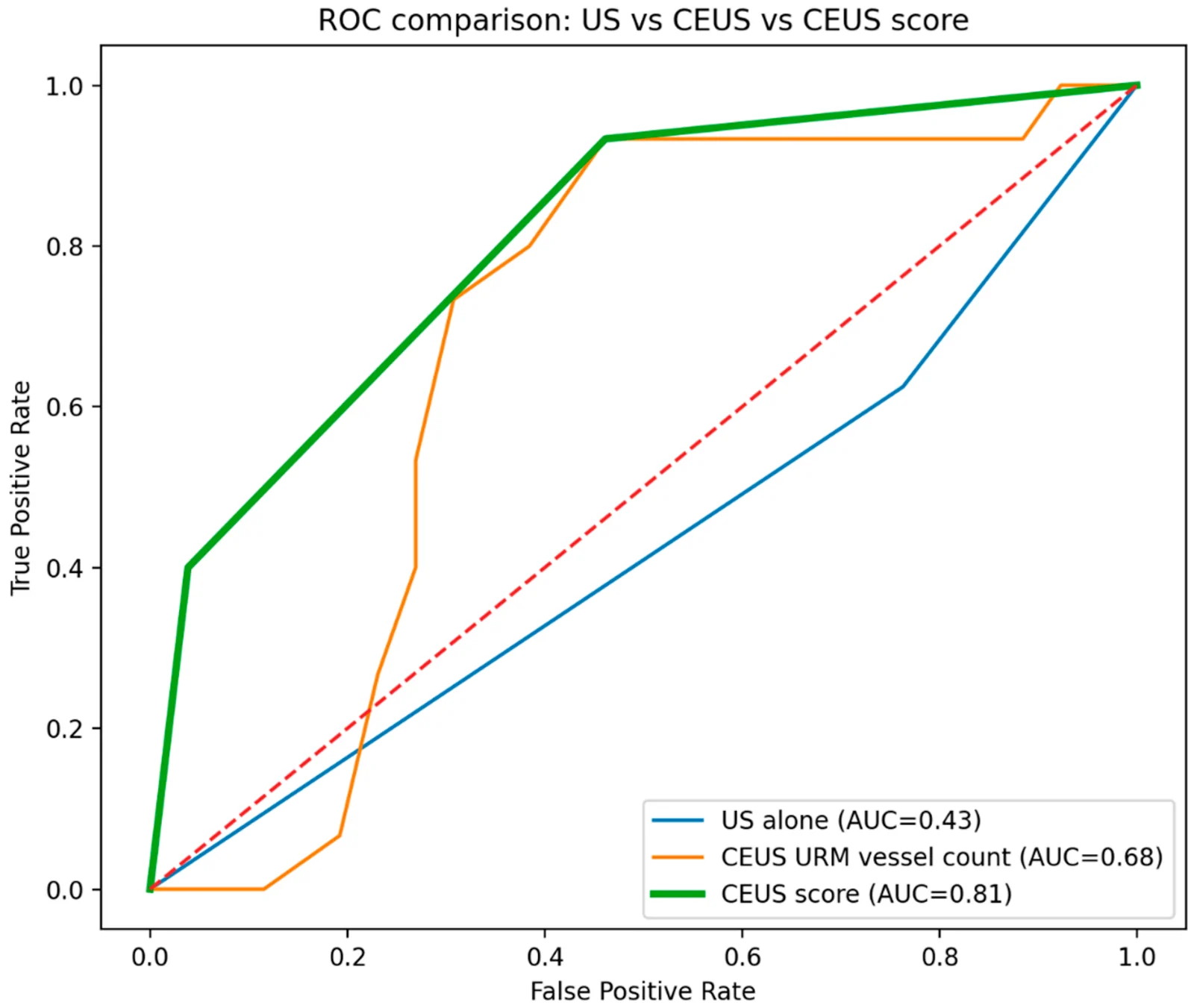
ROC comparison of conventional ultrasound alone, CEUS-URM vessel count, and the CEUS-URM score for predicting axillary lymph node metastasis.

At a low diagnostic threshold, defined as CEUS-URM score ≥ 1, the score showed high sensitivity of 93.3% and an NPV of 93.3%, suggesting potential utility as a rule-out tool for metastatic nodal involvement. At a higher threshold, defined as score = 2, specificity increased to 96.2% and PPV to 85.7%, supporting a potential rule-in role. In Firth penalized logistic regression, each 1-point increase in the CEUS-URM score was associated with increased odds of metastatic lymph node involvement (OR 7.19, 95% CI: 2.04–25.43; *p* = 0.002) (Figure 5).

## 4. Discussion

The present study suggests that CEUS-URM may provide clinically relevant incremental value in one of the most difficult scenarios of axillary ultrasound: lymph nodes with diffuse cortical thickening. In this subgroup, conventional ultrasound often performs sub-optimally because diffuse cortical enlargement may reflect either reactive changes or metastatic infiltration [11,12,13,14,15,16]. The main finding is that a simple CEUS-URM score combining hypervascular vessel count and vascular density outperformed both conventional ultrasound alone and individual quantitative CEUS-URM parameters, reaching an AUC of 0.81. This supports the hypothesis that microvascular information may improve nodal characterization when conventional grayscale criteria are equivocal [18,19,20,27,28,29,30].

Previous studies have shown that axillary ultrasound performance is highly dependent on the morphologic criteria used [11,12,13,14,15,16]. Focal cortical thickness and complete loss of the fatty hilum are generally considered more suspicious than diffuse cortical thickening [12,13,15,16]. In the classification proposed by Bedi et al., focal hypoechoic cortical thickness and completely hypoechoic nodes were considered suspicious, whereas generalized cortical thickness was more problematic and associated with false-negative findings. Other studies have also shown that changing the cortical thickness threshold can markedly affect diagnostic performance; for example, using 3 mm as a threshold increases sensitivity but substantially reduces specificity, while higher thresholds improve specificity at the cost of sensitivity [11,13,16]. These findings support the rationale of the present study: diffuse cortical thickening alone is not sufficiently specific, and additional functional or microvascular information may be needed [31].

The problem is not limited to ultrasound morphology alone. Ultrasound-guided sampling has high specificity when positive, but false-negative results may occur, particularly in small lymph nodes, nodes with limited metastatic involvement, or nodes with relatively thin cortex and enlarged fatty hilum. Ewing et al. reported that smaller lymph nodes, limited nodal replacement, sentinel nodes below 1 cm, and cortical thickness below 3.5 mm were associated with a higher risk of false-negative biopsy. Conversely, false-positive ultrasound findings may occur because cortical thickening can also be reactive or inflammatory [10,14,15,16]. This explains why a technique that visualizes intranodal microvascular organization may be useful: CEUS-URM could help identify malignant angiogenic patterns that are not captured by cortical thickness alone. Similar efforts to improve axillary staging through quantitative and multimodal imaging have also been explored using MRI relaxometry, supporting the broader relevance of functional imaging biomarkers in predicting axillary metastasis [32,33].

The present findings are also consistent with the broader literature on CEUS and super-resolution ultrasound. CEUS has been increasingly investigated for axillary and sentinel lymph node assessment in breast cancer, with recent systematic reviews suggesting high diagnostic potential [18,19,20,21,22,23,24,25,26]. More recently, super-resolution ultrasound has been applied to intranodal lymphatic sinus imaging for prediction of sentinel lymph node metastasis in breast cancer, supporting the feasibility of microvascular and microstructural nodal assessment beyond conventional ultrasound [27]. The present study extends this concept to a clinically challenging subgroup: axillary lymph nodes with diffuse cortical thickening, where conventional ultrasound is particularly limited.

Previous work using URM technique has shown that microbubble localization and tracking can provide visualization of tissue microvasculature beyond the conventional diffraction-limited resolution of ultrasound, able to depict approximately 10-µm-scale microvascular structures in vivo [28,29]. In vivo experimental work has also demonstrated the feasibility of three-dimensional super-resolution ultrasound imaging of lymph node vasculature using microbubbles [30]. Taken together, these data support the biological and technical plausibility of CEUS-URM for assessing nodal microvascular architecture in breast cancer.

From a clinical perspective, CEUS-URM could be used as an adjunct rather than a replacement for standard axillary ultrasound or pathological verification. This is particularly relevant in the current context of axillary management, where accurate nodal staging remains essential despite progressive de-escalation of axillary surgery and increasing reliance on sentinel lymph node biopsy strategies [3,4,5,6,7,8,9,32]. A low CEUS-URM score threshold may be useful as a rule-out tool, given the high sensitivity and negative predictive value observed in this cohort. Conversely, a higher threshold may improve specificity and help select patients who should undergo biopsy or more aggressive axillary management. Future work should validate these thresholds in larger cohorts and determine whether CEUS-URM can reduce unnecessary biopsies, improve biopsy targeting within the most suspicious cortical region, or decrease false-negative sampling by directing the needle toward hypervascular microvascular areas. Studies have reported that contralateral nodal cortical thickness may aid the identification of suspicious nodes, a parameter that could also be incorporated into URM assessment.

Several limitations should be acknowledged. First, the CEUS-URM cohort was relatively small; with only 15 metastatic nodes, so the estimates—particularly the 100% sensitivity/zero false-negative cell—have wide confidence intervals and are optimistic. The score was derived and tested on the same data, providing internal validation by bootstrap (optimism-corrected AUC 0.77) rather than external validation, and no independent cohort was available; these findings are therefore exploratory and hypothesis-generating. Second, this was a single-center study using a specific ultrasound platform, which may limit generalizability of the findings to other imaging systems or CEUS operators. Third, the US-only and CEUS-URM groups were consecutive rather than randomized paired cohorts, and therefore direct comparison between conventional ultrasound and CEUS-URM should be interpreted with caution. Fourth, interobserver and intraobserver agreement for CEUS-URM measurements were not formally assessed; URM quantification depends on region-of-interest placement and operator experience, and formal reproducibility testing is a priority for future work. Fifth, the score thresholds were data-driven and require external validation. Larger prospective multicenter studies are needed to validate CEUS-URM thresholds, assess reproducibility, and determine whether this technique improves clinical decision-making in axillary staging of breast cancer.

## 5. Conclusions

In axillary lymph nodes with diffuse cortical thickening—where conventional ultrasound is least reliable—a simple CEUS-URM score integrating microvascular vessel count and vascular density showed improved, though preliminary, discrimination between benign and metastatic nodes, with potential rule-out (low threshold) and rule-in (high threshold) roles. Because these findings were derived and tested in the same small, single-center cohort, they should be regarded as hypothesis-generating and require validation in larger, external, within-patient paired studies before clinical adoption.

## Figures and Tables

**Table 1:** Baseline characteristics of the study population.

Variable	US Cohort, *n* = 120	CEUS Cohort, *n* = 41	*p*-Value
**Age, years**			0.284 *
Mean ± SD	48.3 ± 12.1	46.0 ± 11.8	
Median, range	48, 29–78	45, 28–79	
**Tumor size category, *n*** (**%**)			0.999 ^‡^
T1	18 (15.0%)	6 (14.6%)	
T2	58 (48.3%)	20 (48.8%)	
T3	38 (31.7%)	13 (31.7%)	
T4	6 (5.0%)	2 (4.9%)	
**Cortical thickness of the evaluated lymph node, mm**			0.481 *
Mean ± SD	5.3 ± 1.8	5.1 ± 1.4	
Median, range	5.0, 3.1–12.0	5.0, 3.1–8.0	
**Histological Nottingham grade, *n*** (**%**)			0.836 ^‡^
G1	11 (9.2%)	4 (9.8%)	
G2	77 (64.2%)	28 (68.3%)	
G3	32 (26.7%)	9 (22.0%)	
**Molecular subtype, *n*** (**%**)			0.196 ^‡^
HR+/HER2−	82 (68.3%)	34 (82.9%)	
HR+/HER2+	22 (18.3%)	2 (4.9%)	
HR−/HER2−	12 (10.0%)	4 (9.8%)	
HR−/HER2+	4 (3.3%)	1 (2.4%)	

* Mann–Whitney U test; ^‡^ chi-square test or Fisher–Freeman–Halton exact test, as appropriate.

**Table 2:** CEUS-URM quantitative parameters in benign and metastatic axillary lymph nodes.

Metric	URM Vessel Count	URM Mean Distance	URM Max Diameter	DV Mean Density	PI (%)	PI (Mean)	PI (Sum)
Benign n	26	26	26	26	26	26	26
Benign median (IQR)	7.00 (3.00–11.50)	345.92 (262.53–481.71)	361.00 (220.28–460.64)	4.24 (2.76–10.62)	5.60 (0.83–18.80)	312.16 (110.88–542.29)	318.53 (116.11–552.30)
Malignant n	15	15	15	15	15	15	15
Malignant median (IQR)	11.00 (9.50–12.50)	332.85 (271.62–358.05)	440.17 (269.27–581.48)	6.49 (3.19–14.50)	16.42 (2.60–25.31)	244.69 (111.02–552.39)	257.58 (116.88–558.91)
Direction	malign > benign	benign > malign	malign > benign	malign > benign	malign > benign	malign > benign	malign > benign
Effect size r	−0.298	0.150	−0.182	−0.220	−0.182	−0.063	−0.068
*p*-value	0.0575	0.3433	0.2500	0.1633	0.2500	0.6947	0.6748
Holm p (all)	0.4027	1.0000	1.0000	0.9798	1.0000	1.0000	1.0000
Holm p (primary)	0.2301	NA	NA	0.4899	0.5000	0.6947	NA
ROC AUC	0.681	0.409	0.610	0.633	0.610	0.538	0.541

DV = density vessel, PI = perfusion index, NA—Not applicable.

**Table 3:** Diagnostic performance of CEUS-URM for detecting metastatic axillary lymph nodes.

Metric	Estimate	95% CI (Exact)	95% CI (Bootstrap)
Sensitivity	100%	78.2–100%	100–100%
Specificity	66.7%	46.0–83.5%	48.1–84.0%
PPV	62.5%	40.6–81.2%	42.3–81.8%
NPV	100%	81.5–100%	100–100%

PPV = positive predictive value, NPV = negative predictive value.

## Data Availability

The original contributions presented in this study are included in the article. Further inquiries can be directed to the corresponding author.
